# Forecasting of Lung Cancer Incident Cases at the Small-Area Level in Victoria, Australia

**DOI:** 10.3390/ijerph18105069

**Published:** 2021-05-11

**Authors:** Win Wah, Rob G. Stirling, Susannah Ahern, Arul Earnest

**Affiliations:** 1 Department of Epidemiology and Preventive Medicine, School of Public Health and Preventive Medicine, Monash University, Melbourne 3004, Australia; Susannah.Ahern@monash.edu (S.A.); Arul.Earnest@monash.edu (A.E.); 2 Department of Allergy, Immunology & Respiratory Medicine, Alfred Health, Melbourne 3004, Australia; r.stirling@alfred.org.au; 3 Department of Medicine, Monash University, Melbourne 3168, Australia

**Keywords:** lung cancer, forecast, Bayesian, spatio-temporal, age-period-cohort

## Abstract

Predicting lung cancer cases at the small-area level is helpful to quantify the lung cancer burden for health planning purposes at the local geographic level. Using Victorian Cancer Registry (2001–2018) data, this study aims to forecast lung cancer counts at the local government area (LGA) level over the next ten years (2019–2028) in Victoria, Australia. We used the Age-Period-Cohort approach to estimate the annual age-specific incidence and utilised Bayesian spatio-temporal models that account for non-linear temporal trends and area-level risk factors. Compared to 2001, lung cancer incidence increased by 28.82% from 1353 to 1743 cases for men and 78.79% from 759 to 1357 cases for women in 2018. Lung cancer counts are expected to reach 2515 cases for men and 1909 cases for women in 2028, with a corresponding 44% and 41% increase. The majority of LGAs are projected to have an increasing trend for both men and women by 2028. Unexplained area-level spatial variation substantially reduced after adjusting for the elderly population in the model. Male and female lung cancer cases are projected to rise at the state level and in each LGA in the next ten years. Population growth and an ageing population largely contributed to this rise.

## 1. Introduction

Lung cancer has been the most commonly diagnosed cancers and is the leading cause of cancer deaths worldwide [1]. Time delays between the date of cancer diagnosis, registry notification, receipt of all relevant notifications to the cancer registry, and time required to verify cases at the registry lead to a lag of up to one year in publishing incidence data in the routinely published reports [2]. Projecting lung cancer incidence trends is complex due to changing risk factor profiles over the years and the long latency period between risk factor exposure and lung cancer development. Although lung cancer incidence is known to vary geographically within Australia [3], robust and precise forecasts for small-area units within each state is lacking. Thus, statistical models providing predictions for lung cancer counts for different individual areas are useful to quantify the lung cancer burden fundamental to cancer-control planning, allocation of healthcare resources, and research. 

Despite the significant amount of research on the spatio-temporal evolution of disease mapping risks using Bayesian spatio-temporal models, there is little published literature on applying those models to provide reliable projections of lung cancer counts at the small-area level. Bayesian spatio-temporal models borrow strengths from neighbouring areas to improve estimates of areas with sparse counts or a small population [4]. Thus, this study aims to forecast lung cancer counts at the local government areas (LGA) level over the next ten years (2019–2028) in Victoria, Australia. Victoria is the second most populous state in Australia, with an estimated residential population of 6.6 million in 2020 [5]. 

## 2. Methods

### 2.1. Data

This study included 46,297 incident lung cancer cases (lung, bronchus, trachea, ICD-10-C33-34) diagnosed among Victorian residents between 2001 and 2018 from the Victorian Cancer Council Registry (VCR) [2]. All diagnosed cancer cases, except skin cancer, are notifiable to the VCR by law. Patients were assigned to 79 LGAs based on their residential postcode at diagnosis. 

Annual male and female resident populations by different age groups at the LGA levels (2001–2019) were obtained from the Australia Bureau of Statistics (ABS) [6]. Projected population counts at the LGA level were extracted from the Department of Environment, Land and Water planning, which incorporates population growth using fertility, mortality, and migration rates [7]. 

In our models, we included covariates such as the proportion of men and women currently smoking [8], proportion of the population who were elderly (≥65) [6], pollution categories [9], and socio-economic disadvantage status [10] reported at the LGA level. The index of relative socio-economic disadvantage (IRSD) was used as a surrogate indicator of area-level socio-economic status (SES) [10]. An area with a low IRSD score indicates a high proportion of relatively disadvantaged people in the LGA. Pollution category data (high, medium, low, no polluting facilities) were obtained from the National Pollutant Inventory [9]. We categorized the proportion of current male and female smokers, elderly population, and IRSD into quartiles and stratified remoteness into two remoteness areas: metropolitan/major cities versus regional/remote [11]. 

### 2.2. Statistical Analysis

All analyses were stratified by gender because lung cancer incidence is known to vary by gender [12]. Through direct internal standardization using the Victorian census population data, we calculated the age-standardized expected counts of the particular LGA in the observed years by multiplying with the age-standardized incidence rates of the specific LGA and year and the number of the population of the particular LGA and year [13]. The age-standardized incidence rates were derived by dividing the observed lung cancer counts by the population in each age category, LGA, and year and multiplying with the population’s proportion within the particular age category (population weights). 

**Figure 1:**
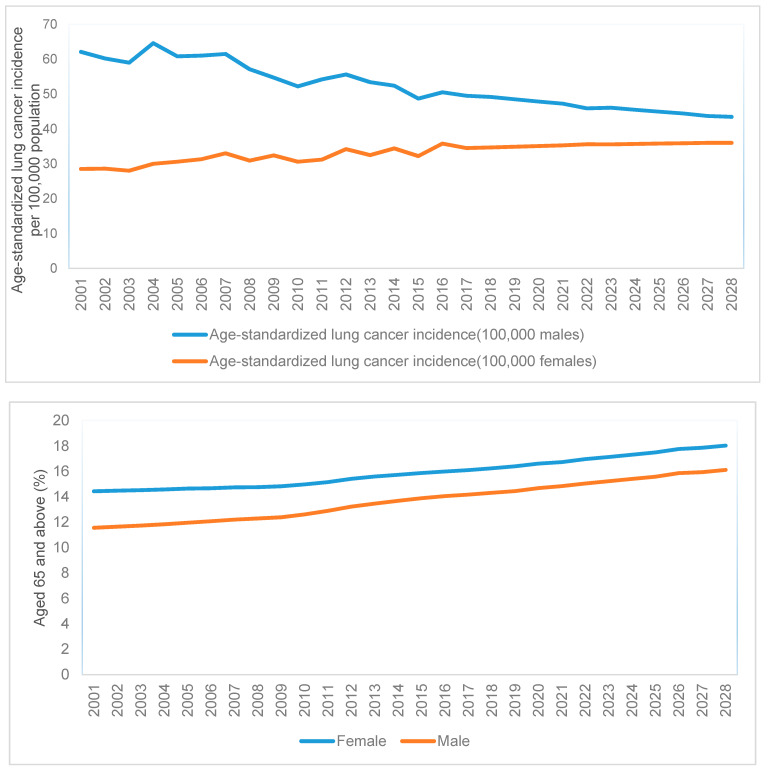
Line plot of annual lung cancer incidence and proportion of the elderly population.

The age-standardized expected counts for each LGA in the projected years were obtained by multiplying the projected population in each age category, LGA, and year with the estimated age-specific incidence of that year derived from Age–Period–Cohort (APC) modelling. An APC model that extrapolates the age, period, and cohort trends into the future has been shown empirically to perform well in predicting cancer incidence [14]. An APC model with a power link function was fitted, assuming the number of cases followed a Poisson random variable with the logarithm of the person-years at risk specified as an offset [14]. Figure 1 presents the age-standardized incidence rates from the data and derived from the APC model, using direct standardisation with the Australian 2001 population [15,16].

*Y_it_* ~ Poisson(*µ*_it_)

log*µ*_it_ = log *e*_it_ + *α* + *φ*_i_ + *ν*_i_ + *t*_t_ + *t*_t_^2^

The Bayesian spatio-temporal model takes the age group distribution in each area and year into account through the derived expected counts in each area and year (*e_it_*) [17,18,19,20]. In this model, the observed disease count in area *i* and year *t* (*Y_it_*) is assumed to follow a Poisson distribution with relative disease risk (*µ*_it_). The log relative risk of lung cancer in each area and year (*µ*_it_) was modelled as a function of the intercept (*α*), spatially structured (*φ*_i_) and unstructured (*ν*_i_) random-effects, and linear (*t*_t_) and quadratic (*t_t_*^2^) temporal terms. The priors for the standard deviation of the precision estimates were set a uniform distribution (0.01 to 10). Those for the means were assigned to a normal distribution with a standard deviation covering a wide range of values. The intercept term and the coefficients for the time period indicator and unstructured spatial variation were assigned vague normal priors. Structured spatial variation was assumed to follow an intrinsic CAR (conditional autoregressive) prior that neighbours were assigned based on geographically adjacent boundaries [17]. This CAR prior allows smoothing of estimates in each LGA towards the mean risk in the neighbouring LGA and improves estimates where the expected counts are low [17].

*Y_it_* ~ Poisson(*µ*_it_)

log*µ*_it_ = log*e*_it_ + *α* + *φ*_i_ + *ν*_i_ + *t*_t_ + *ω_t_*

We compared this model with the autoregressive temporal terms, including the first-order random walk (*ω_t_*), to predict future disease rates based on past trends. It assumes that each year incidence depends on the preceding year incidence to enable correlation between consecutive years [21]. 

We ran two Markov chain Monte Carlo chains starting from different initial values in each model using MultiBugs software [22]. Supplementary Material-1 presents details of the Bayesian spatio-temporal model. We calculated the unexplained area-level variation associated with geographical location explained by the model [23]. Among the competing models, we chose the best-fitting model based on the deviance information criteria (DIC) [24,25]. 

To evaluate the forecasting models’ performance, we used the first 13 years of data (2001–2013) as a training dataset to forecast for the remaining validation dataset (2014–2018). We assessed the predictive performance of the selected model using prediction accuracy measures such as root mean squared error (RMSE), mean absolute error (MAE), and mean absolute percentage error (MAPE) within the training and validation datasets [26]. We set the model with the highest prediction accuracy (lowest error measures) based on the validation dataset for out-of-sample predictions. 

## 3. Results 

There were 27,555 male (59.52%) and 18,742 female (40.48%) cases recorded between 2001 and 2018. Rates of male and female lung cancer incidence were observed to increase with age. Overall, lung cancer incidence trends showed a clear differential pattern between men and women (Figure 1). In males, a fluctuating downward trend in age-standardized rates from 62.1 in 2001 to 49.2 in 2018 was observed, contrasting with increasing rates in women, from 28.5 in 2001 to 34.7 in 2018. Projected age-standardized incidence rates from APC models showed a slightly decreasing trend in males and an increasing trend in females after 2018. There were higher proportions of females than males in the older age groups (≥65) (Figure 1). The spatio-temporal model with linear and quadratic temporal terms had lower DIC and better model fit for male and female lung cancer incidence (Table 1). Thus, this model was selected for further analyses comparing different models with covariates using training and validation data (Table 2). The models without any covariates showed better prediction accuracy than those with other covariates in the validation dataset. Hence, this simple, parsimonious model was chosen for out-sample predictions of male and female lung cancer counts. 

**Figure 2:**
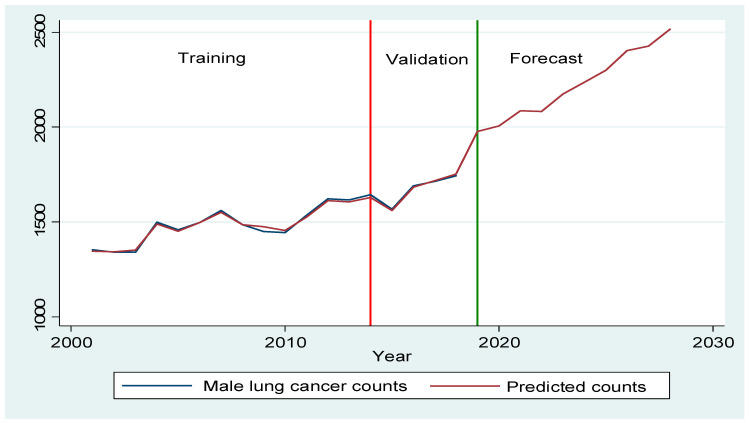

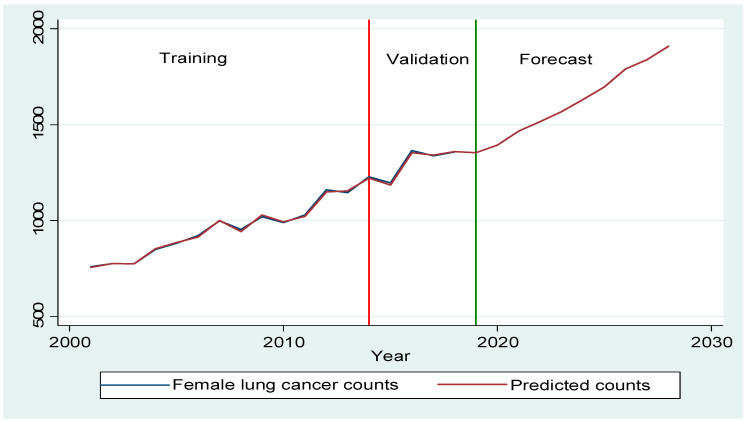
Comparison of observed and predicted male and female lung cancer counts per year.

**Figure 3:**
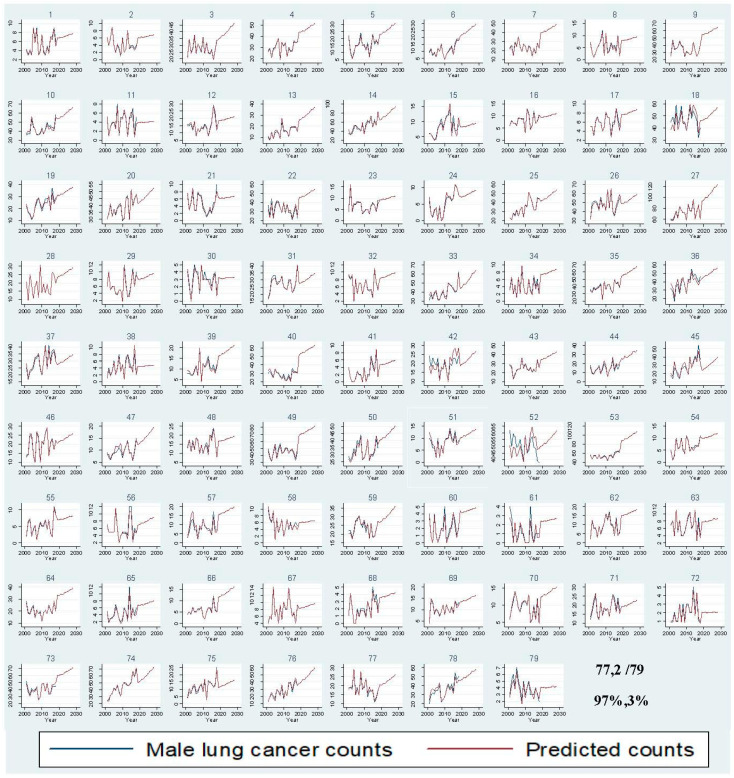
Comparison of observed and predicted male lung cancer counts per year by Local Government Areas.

**Figure 4:**
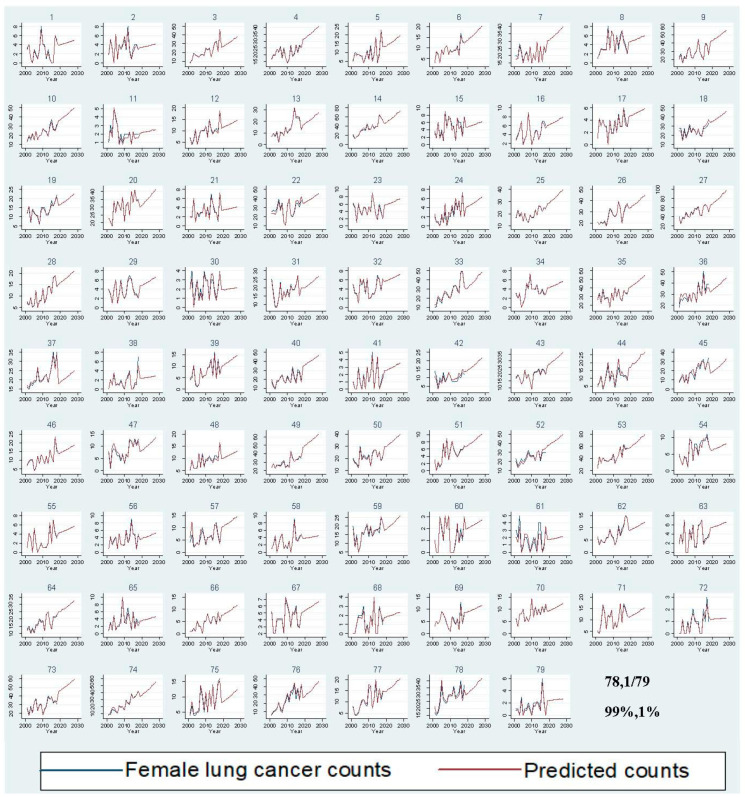
Comparison of observed and predicted female lung cancer counts per year by Local Government Areas.

**Figure 5:**
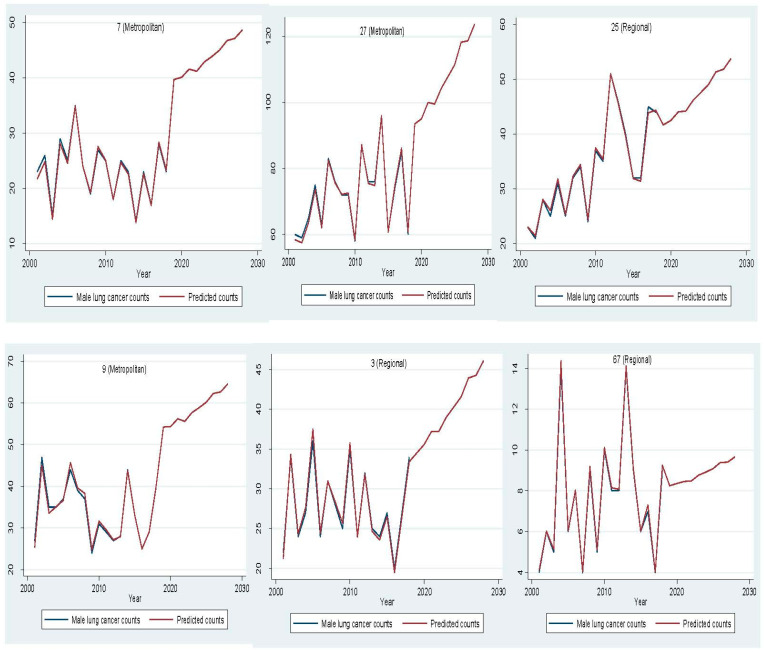
Comparison of observed and predicted male lung cancer counts per year in selected Local Government Areas by remoteness category.

**Figure 6:**
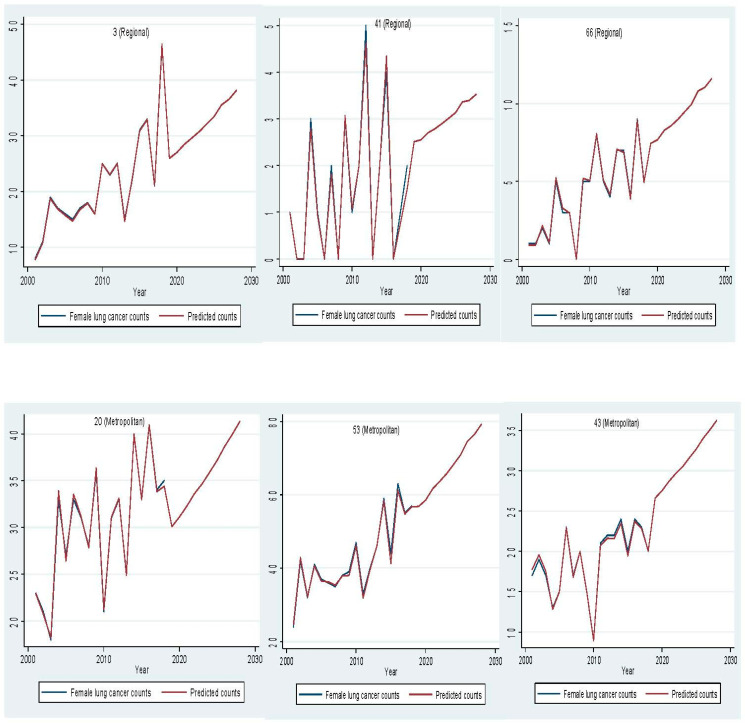
Comparison of observed and predicted female lung cancer counts per year in selected Local Government Areas by remoteness category.

In 2018, lung cancer incidence increased by 28.82% from 1353 to 1743 cases for men and by 78.79% from 759 to 1357 cases for women compared to 2001 (Figure 2). Between 2019 and 2028, male lung cancer incidence is expected to reach 2515 cases, with a 44% increase, and female lung cancer incidence is projected to rise to 1909 cases, with a 41% increase (Supplementary Table S1). Out of 79 LGAs, most LGAs will have a rising trend for male (77.97%) and female (78.99%) lung cancer counts between 2019 and 2028 (Figure 3 and Figure 4). A stable trend of each LGA was defined as less than a 5% increase from the average of previous years. Around 3% and 1% of LGA will have a stable trend among men and women in the next ten years. Figure 5 and Figure 6 display the observed and predicted lung cancer counts in LGA by remoteness category. These LGA were selected to represent the distribution of metropolitan/regional status. The high proportion of the elderly population at the LGA level was significantly associated with an increased risk of lung cancer. The unexplained area-level spatial variation decreased from 92% to 19% among men and from 82% to 19% among women after adjusting for the area-level proportion of current smokers and the elderly. Plots of standardized residuals versus predicted counts showed no obvious outliers of LGA, based on the definition of standardized residuals of >2 or <−2 [27] (Supplementary Figure S1).

## 4. Discussion

This study provided predictions of male and female lung cancer cases that would continue to rise at the state level and in each LGA in the next ten years. Lung cancer incidence is projected to increase by 44% and 41% among men and women in the next ten years. The higher proportion of the elderly population at the LGA level was significantly associated with higher lung cancer risk. Including linear and non-linear components in some LGA and lower DIC values reflected better model fit on this study data with the associated spatio-temporal model with linear and quadratic temporal terms. 

The predicted increase found in this study possibly reflects the demographic changes that Victoria will experience in the next decade, notably population growth and ageing. This study included age-standardized expected counts and population projections to take into account changes in the ageing and population size. However, the age-adjusted rates do not show the demographic changes in the population. Predicting the future number of incident cases rather than predicting age-standardized rates would be more beneficial to quantify the impact of cancer burden for health planning purposes. 

Selecting an appropriate projection method mainly relies on data availability and the purpose of the forecasts. These methods range from the present state method assuming the current rate remains unchanged into the future to more complex statistical models of historical trends, including APC and generalized linear mixed model (GLMM) [28].

The APC model is the most commonly used projection method for lung cancer incidence and remains appropriate for long-term projections [28]. Period effects can represent screening, diagnostic, and treatment factors that led to lung cancer incidence variation across all age groups. In contrast, the cohort effect can reflect smoking histories and tobacco uptake and cessation rates influenced by societal and peer factors from generation to generation. This study used two-step forecasting methods with the APC model to derive age-adjusted expected counts first. It then included those projected expected counts in the Bayesian spatio-temporal GLMM model to predict the validation and out-sample data while incorporating possible area-level risk factors. We acknowledge the uncertainty in measurement error due to the two-step process. Other methodological papers derived expected counts differently by multiplying the projected population in each age group, area, and year with the age-specific rates corresponding to the entire study region and period of observed data [18]. 

Other forecasting studies on lung cancer incidence did not consider the spatial dimension. The trend of predicted male and female lung cancer counts and age-standardized rates at the annual level was in line with projected rates in other studies [16,29,30,31,32,33]. Consistently, new cases are projected to increase among men despite a slight decrease (1%) in age-standardized rates after 2018. Other studies also reported increasing counts and age-standardized rates among females [16,29,30,31,32,33].

Given the established association between tobacco smoking and lung cancer risk, projections of lung cancer incidence are also crucial for evaluating the existing tobacco control program’s effectiveness. In the 1970s, after the initial publicity relating to smoking’s effect in Australia, smoking among men declined, but it increased among women [34]. With the introduction of tobacco control policies in the late 1990s, smoking prevalence has decreased in both sexes since 2001 (Supplementary Figure S2) [34]. In 2019, around 12.9% of Australians aged 18 years≥ smoked daily or weekly compared to 22.2% in 2001 [34]. Different tobacco consumption histories could explain variation in lung cancer incidence trends between men and women. Declines in smoking among men in the second half of the 20th century have subsequently been reflected in the downward trend of lung cancer incidence in men after 2001, with a time lag of around 20 years [34]. Much later uptake of tobacco smoking in those earlier years among women could explain the rising proportion of lung cancer cases between 2001 and 2018 and a projected increasing trend [34].

This study only adjusted for the area-level covariates due to the lack of individual-level smoking, genetic, and environmental exposure data in the VCR data. The area-level elderly population significantly reduced unexplained area-level spatial variation. However, those areal covariates did not improve the models’ prediction accuracy as they may not fully explain the variation, and these are areal measures compared to individual patient-level data. Though lung cancer is primarily caused by tobacco smoking, studies have reported a two-fold rise in the proportion of non-smokers with lung cancer in recent years [35]. Lung cancer in non-smokers is almost exclusively non-small cell lung cancer, with non-smoking women at higher risk [35]. This shift in aetiology might support an increase in lung cancer counts, particularly among females, over the years in this study, despite a decline in smoking prevalence.

The growth of the elderly population has been consistently greater for women than men and contributed to higher lung cancer incidence. In men, the increase in lung cancer incidence could be mainly due to population growth and ageing. In women, estrogens and other female hormones could have influenced the increased risk of non-smoking related lung cancer independent of age and smoking status [35,36].

Population-based lung cancer screening has not been adopted in Australia due to insufficient evidence on benefits [37]. Current Australian guidelines recommend lung cancer screening in those 55–74 years of age who smoke at least 30 packs per year, current smokers, or those who have quit smoking in the past 15 years [37]. However, this applicability in Aboriginal Torres Straits Islander populations remains to be confirmed.

This population-based study evaluated the model performance using training and validation data and the goodness-of-fit and prediction accuracy measures. We used Bayesian models to account for different sources of uncertainty and different spatial and temporal patterns. Bayesian spatio-temporal models borrow strength across space and time to do spatio-temporal smoothing and reduce the effect of random measurement error [21].

Limitation of the models includes caution in extrapolating model predictions beyond the range of data. Additionally, the models did not account for spatio-temporal interactions to allow for area-specific varying disease risk trends. The models are only useful for predicting aggregate level counts but not for individual-level inference, thus leading to a potential risk of ecological bias. Our projections assume that the effects of age, period, and cohort components will remain unchanged into the future. However, our assumption is supported by similar annual patterns predicted for lung cancer in other countries or Australia [16,29,30,31,32,33]. Impact on lung cancer screening and healthcare utilization due to the COVID-19 pandemic may result in decreased numbers of lung cancer diagnosis during 2020–2021. Our models would then be overestimating lung cancer counts, which is a limitation we acknowledge. These forecasts assume no sudden changes in health care utilisation due to natural disaster or pandemics such as COVID-19. Thus, caution is required with interpretations on projection estimates due to the potential impact of the COVID-19 pandemic. Projections also need to consider time lag assessment of disease rates (>10 years) between changes in risk factors or tobacco-control policies. Additionally, this study did not attempt to model the future impact of tobacco control interventions.

Market research company Euromonitor predicts the decline in the prevalence of smoking in Australia is set to continue [38]. Even if smoking rates continue to decline, the effects of past smoking are expected to continue to impact lung cancer. The increase in cancer cases in women and men reinforces the need to continue and strengthen the tobacco control measures and expand efforts in early cancer detection provided by screening. These predictions at the LGA level help inform the existing and future burden of cancer, the impact of public health policies and strategies on cancer and decision-making for efficient resource allocation of cancer detection, prevention, and treatment in Australia [39,40]. This study identified LGAs with increasing incidence over time, which is useful for resource allocation and policy implementation of lung cancer prevention and control. A geographically-targeted approach could highlight areas needing additional services for managing lung cancer more effectively, such as where to concentrate screening. The models can be applied to spatio-temporal analyses of lung cancer incidence and different diseases in other regions. These projections provide a platform to investigate possible variation in lung cancer incidence for various policy interventions and plan the proper development of health services such as diagnostic, therapeutic, and palliative procedures at the LGA level.

## 5. Conclusions

Lung cancer incidence among men and women is increasing in the next ten years. The number of people with lung cancer in Victoria is likely to increase by 44% in males and 41% in females from 2019 to 2028, driven mainly by population growth and ageing. This study highlights higher lung cancer incidence among females despite a decline in smoking rates and taking population ageing into account. These results have important policy implications in terms of planning healthcare needs and public health spending. The prediction estimates in this study also act as a benchmark to measure the impact of possible lung cancer prevention initiatives.

## Figures and Tables

**Table 1:** Comparison of goodness-of-fit of the Bayesian spatial-temporal models.

**Male Lung Cancer Incidence**	**DIC**
Model A (Linear & quadratic temporal terms)	**4532**
Model B (Autoregressive temporal terms)	4610
**Female lung cancer incidence**	DIC
Model A (Linear & quadratic temporal terms)	**3940**
Model B (Autoregressive temporal terms)	4061

*DIC; Deviance information criterion, Lower DIC indicates better model fit (Bold).*

**Table 2:** Comparison of predictive accuracy of the Bayesian spatio-temporal models.

	Male				Female			
Training (2001–2013)	RMSE	MAE	MAPE	DIC	RMSE	MAE	MAPE	DIC
Model A (Linear & quadratic temporal terms)	1.34	0.71	3.86	4532	0.89	0.44	4.12	3940
Model A + Smoking	1.33	0.71	3.75	4478	0.90	0.45	4.20	3982
Model A + Smoking + RSD	1.34	0.71	3.79	4356	0.89	0.45	4.07	3995
Model A + Smoking + Pollution	1.35	0.73	4.04	4498	0.91	0.46	4.48	3985
Model A + Smoking + Elderly	1.38	0.74	3.85	4578	0.96	0.48	4.19	4031
Model A + Smoking + Elderly + Pollution	1.39	0.75	3.89	4575	0.94	0.48	4.44	4029
**Validation (2014–2018)**	**RMSE**	**MAE**	**MAPE**	**DIC**	**RMSE**	**MAE**	**MAPE**	**DIC**
Model A (Linear & quadratic temporal terms)	**8.75**	**5.87**	**39.57**	**4532**	**6.35**	**4.27**	**31.99**	**3940**
Model A + Smoking	8.80	5.91	40.26	4478	6.40	4.32	33.17	3982
Model A + Smoking + RSD	8.81	5.89	40.06	4356	6.40	4.34	33.04	3995
Model A + Smoking + Pollution	8.80	5.92	40.57	4498	6.41	4.36	33.83	3985
Model A + Smoking + Elderly	8.80	5.93	41.61	4578	6.40	4.34	33.87	4031
Model A + Smoking + Elderly + Pollution	8.81	5.94	41.90	4575	6.43	4.37	33.84	4029

*RMSE; Root mean squared error, MAE; Mean absolute error, MAPE; Mean absolute percentage error, DIC; Deviance information criterion. Lower values of these measures indicate better prediction accuracy (Bold).*

## Data Availability

Restrictions apply to the availability of these data. Data was obtained from Victorian Cancer Registry and are available with the permission of Victorian Cancer Registry.

## Supplementary Materials

The following are available online at https://www.mdpi.com/article/10.3390/ijerph18105069/s1, Supplementary Material-1 Description of Bayesian spatial-temporal model, Table S1: Comparison of observed and predicted male and female lung cancer counts per year with 95% credible intervals, Figure S1: Plot of standardized residuals versus predicted male and female lung cancer counts, Figure S2: Prevalence of regular smokers for all Australians and by gender (1980–2019).

## Abbreviation

ABS	Australia Bureau of Statistics
APC	Age–Period–Cohort model
CAR	Conditional autoregressive
DIC	Deviance Information Criteria
ICD	International Classification of Diseases
IRSD	Index of relative socio-economic disadvantage
LGA	Local Government Areas
MAPE	Mean absolute percentage error
MSE	Mean squared error
RMSE	Root mean squared error
SES	Socio-economic status
VCR	Victorian Cancer Registry
