# Supplementary material for: Forecasting of Lung Cancer Incident Cases at the Small-Area Level in Victoria, Australia
Source: Int J Environ Res Public Health. 2021 May 11;18(10):5069. doi: 10.3390/ijerph18105069 (PMC8151486; doi:10.3390/ijerph18105069)
Supplement: Supplementary file 1 [file ijerph-18-05069-s001.zip › ijerph-1196688-supplementary.pdf]

Supplementary Material-1 Description of Bayesian spatial-temporal model
Supplementary Table-S1 Comparison of observed and predicted male and female lung cancer counts per year with 95% credible intervals
Supplementary Figure-S1 Plot of standardized residuals versus predicted male and female lung cancer counts
Supplementary Figure-S2 Prevalence of regular smokers for all Australians and by gender (1980–2019)

Supplementary Material-1 Description of Bayesian spatial-temporal model

Spatial-temporal model with linear and quadratic temporal terms

$Y_{it} \sim \text{Poisson}(\mu_{it})$

$\log \mu_{it} = \log e_{it} + \alpha + \varphi_i + v_i + t_t + t_t^2$

The bayesian spatial-temporal model takes the age group distribution in each area and year into account through the derived expected counts in each and year (e_{it})[1-4]. In this model, the observed disease count in area i and year t (Y_{it}) is assumed to follow a Poisson distribution with relative disease risk (μ_{it}). The log relative risk of lung cancer in each area and year was modelled as a function of the intercept (α), spatially structured (φ_i) and unstructured (v_i) random effects and linear (t_t) and quadratic (t_t^2) temporal terms. The priors for the standard deviation of the precision estimates were set a uniform distribution (ranging from 0.01 to 10). Those for the means were assigned to a normal distribution with a standard deviation covering a wide range of values. The intercept term, coefficients for the time period indicator and unstructured spatial variation were assigned vague normal priors. Structured spatial variation was assumed to follow an intrinsic CAR (conditional autoregressive) prior that neighbours were assigned based on geographically adjacent boundaries[1]. This CAR prior allows smoothing of estimates in each LGA towards the mean risk in the neighbouring LGA and improves estimates where the expected counts are low[1].

Spatial-temporal model with autoregressive temporal terms

$Y_{it} \sim \text{Poisson}(\mu_{it})$

$\log \mu_{it} = \log e_{it} + \alpha + \varphi_i + v_i + t_t + \omega_t$

We compared the Bernardinelli model with the autoregressive temporal terms, including the first-order random walk (ω_t), to predict future disease rates based on past trends. It assumes that each year incidence depends on the preceding year incidence to enable correlation between consecutive years[5]. We ran two Markov Chain Monte Carlo chains starting from different initial values in each model using MultiBugs software[6]. Model convergence was relatively good, with convergence observed at approximately 10,000 iterations. The first 10,000 samples were discarded as burn-in, and further 90,000 iterations were used to calculate the statistics. Convergence was assessed by Gelman-Rubin diagnostic plots and autocorrelation plots[7]. We also evaluated whether the Monte Carlo standard error estimates of the estimated parameters were less than 5% of the posterior standard deviation. We calculated the unexplained area-level variation associated with geographical location[8].

Among the competing models, we chose the best-fitted model based on deviance information criteria (DIC)[9, 10]. We considered the model selection criterion to suggest models within 1-2 DIC units of the best model (lowest DIC) as strongly supported, 3-7 as having less support, >7 as no support.

References:

1. Bernardinelli L, Clayton D, Pascutto C, et al. Bayesian analysis of space—time variation in disease risk. Statistics in medicine. 1995;14(21-22):2433-43.
2. Corpas-Burgos F, Martinez-Beneito MA. An Autoregressive Disease Mapping Model for Spatio-Temporal Forecasting. Mathematics. 2021;9(4):384.
3. Beard JR, Earnest A, Morgan G, et al. Socioeconomic disadvantage and acute coronary events: a spatiotemporal analysis. Epidemiology. 2008:485-92.
4. Wah W, Ahern S, Evans S, et al. Geospatial and temporal variation of prostate cancer incidence. Public Health. 2021;190:7-15.
5. Bailey T. An introduction to spatial and spatio-temporal modelling of small area disease rates. Spring Course organizado pelos Instituto de Ciências Biomédicas de Abel Salazar, Faculdade de Medicina e Instituto de Saúde Pública da Universidade do Porto. 2008.
6. Goudie RJ, Turner RM, De Angelis D, et al. MultiBUGS: A parallel implementation of the BUGS modelling framework for faster Bayesian inference. arXiv preprint arXiv:170403216. 2017.
7. Brooks SP, Gelman A. Alternative methods for monitoring convergence of iterative simulation. Journal of Computational and Graphical Statistics. 1998;7:434-55.
8. Dasgupta P, Cramb SM, Aitken JF, et al. Comparing multilevel and Bayesian spatial random effects survival models to assess geographical inequalities in colorectal cancer survival: a case study. International journal of health geographics. 2014;13(1):36.
9. Martínez-Bello DA, López-Quílez A, Torres-Prieto A. Bayesian dynamic modeling of time series of dengue disease case counts. PLoS neglected tropical diseases. 2017;11(7):e0005696.
10. Best N, Richardson S, Thomson A. A comparison of Bayesian spatial models for disease mapping. Statistical methods in medical research. 2005;14(1):35-59.

Supplementary table-S1 Comparison of observed and predicted male and female lung cancer counts per year with 95% credible intervals

	**Male**		**Female**	
**Year**	**Observed counts**	**Predicted counts (95% credible intervals)**	**Observed counts**	**Predicted counts (95% credible intervals)**
2001	1353	1345.36(1220.06-1482.82)	759	755.98(675.03-845.51)
2002	1340	1341.7(1219.11-1475.55)	775	775.37(693.53-865.86)
2003	1341	1350.82(1232.59-1478.96)	774	773.83(693.76-862.25)
2004	1498	1488.77(1357.52-1631.4)	850	852.87(766.52-947.84)
2005	1458	1451.55(1326.45-1586.78)	882	885.58(796.5-983.65)
2006	1496	1496.04(1365.32-1637.97)	922	913.89(822.88-1013.92)
2007	1559	1551.06(1417.45-1696.04)	999	1001.05(898.06-1114.77)
2008	1486	1484.65(1353.75-1626.9)	953	943.55(847.86-1048.9)
2009	1449	1474.3(1346.43-1612.84)	1020	1028.62(922.36-1145.84)
2010	1444	1453.89(1325.32-1593.44)	990	993.99(893.98-1103.82)
2011	1535	1524.4(1392.18-1667.64)	1029	1021.18(917.72-1135.15)
2012	1622	1612.9(1473.5-1764.15)	1160	1149.29(1034.29-1275.57)
2013	1616	1604.36(1466.59-1753.53)	1145	1154.93(1037.19-1284.96)
2014	1643	1629.33(1488.42-1782.03)	1229	1221.98(1097.1-1359.39)
2015	1567	1559.08(1422.86-1707.3)	1196	1185.18(1065.9-1316.42)
2016	1691	1683.54(1534.63-1845.71)	1364	1354.89(1219-1504.43)
2017	1714	1718.03(1564.59-1885.22)	1338	1341.2(1203.55-1493.09)
2018	1743	1751.61(1595.74-1921.45)	1357	1359.18(1216.41-1517.26)
2019		1976.49(1789.72-2181.61)		1354.28(1208.78-1516.13)
2020		2006.77(1810.46-2224.05)		1393.8(1238.64-1567.55)
2021		2086.34(1872.72-2324.55)		1468.09(1297.01-1660.88)
2022		2082.71(1857.6-2336.28)		1515.67(1329.32-1727.78)
2023		2174.23(1924.64-2458.88)		1568.7(1363.32-1804.42)
2024		2235.83(1961.33-2552.23)		1631.5(1403.06-1897.05)
2025		2300.51(1997.43-2654.85)		1696.88(1441.72-1997.88)
2026		2403.95(2063.17-2808.59)		1789.93(1499.85-2137.1)
2027		2426.66(2056.24-2873.17)		1838.3(1516.92-2229.49)
2028		2515.05(2101.2-3022.47)		1909.42(1549.17-2355.52)

Supplementary Figure-S1 Plot of standardized residuals versus predicted male and female lung cancer counts

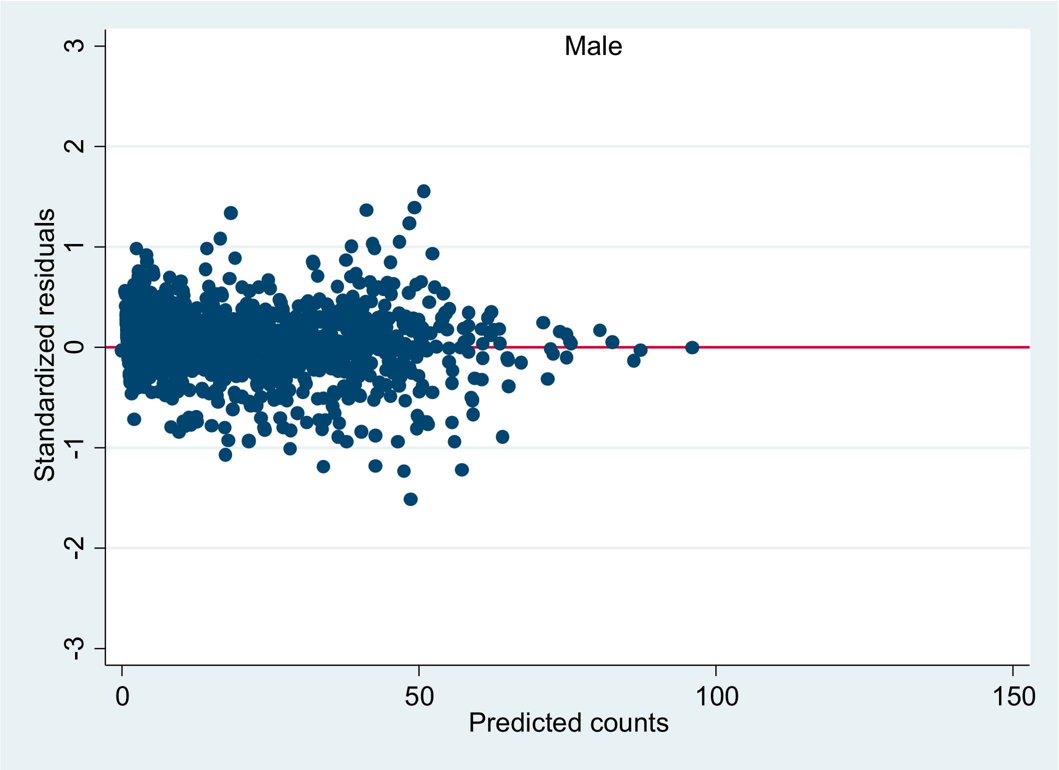

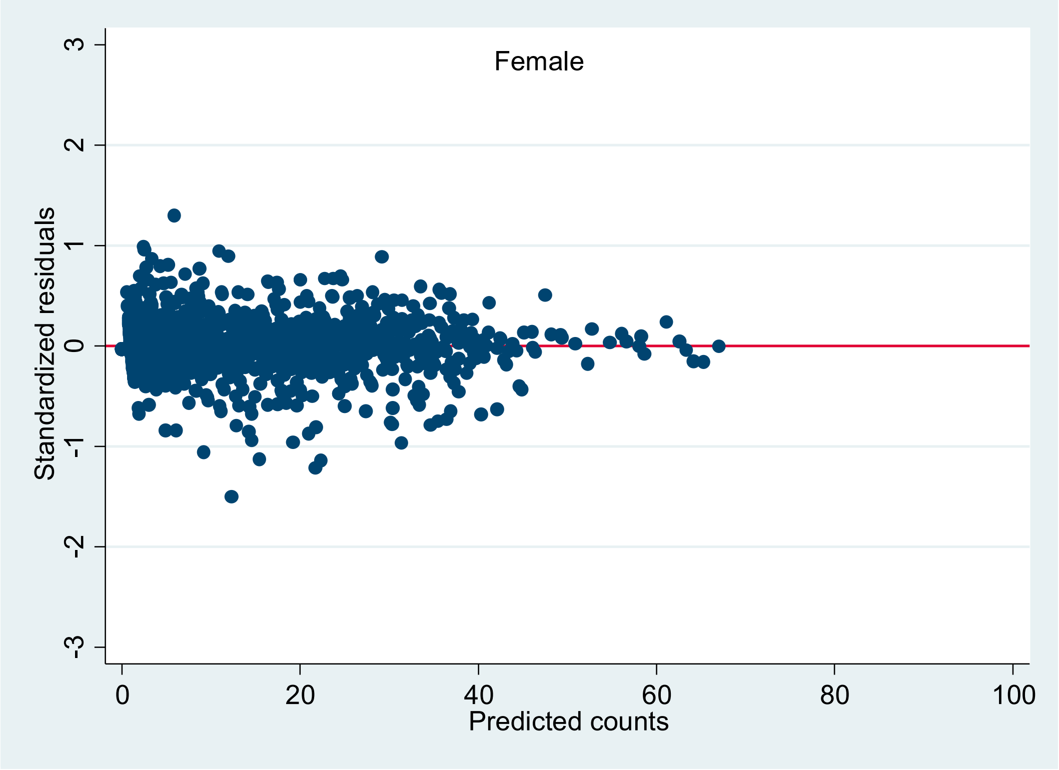

Each point represents each local government area

Standardized residuals = Observed counts - Predicted counts /$\sqrt{\text{Predicted counts}}$

Supplementary Figure-S2 Prevalence of regular smokers for all Australians and by gender (1980–2019)

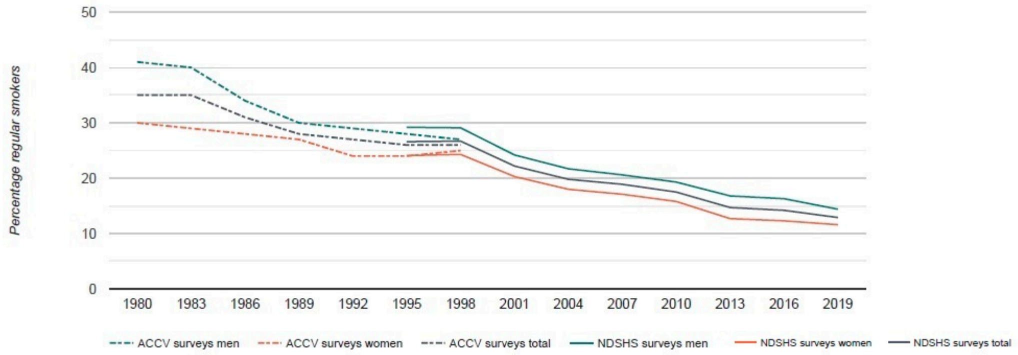

** Anti-Cancer Council of Victoria (ACCV) data includes those describing themselves as 'current smokers' with no frequency specified; National Drug Strategy Household Survey (NDSHS) data includes those reporting that they smoke 'daily' or 'at least weekly'.*

Source Greenhalgh, EM, Bayly, M &Scollo MM. 1.3 Prevalence of smoking—adults. In Greenhalgh, EM, Scollo, MM and Winstanley, MH [editors]. Tobacco in Australia: Facts and issues. Melbourne: Cancer Council Victoria; 2021. Available from https://www.tobaccoinaustralia.org.au/chapter-1-prevalence/1-3-prevalence-of-smoking-adults#:~:text=According%20to%20the%20Australian%20Bureau,aged%2015%20years%20and%20over
